# Supplementary material for: Deciphering Winter Sprouting Potential of Erianthus procerus Derived Sugarcane Hybrids under Subtropical Climates
Source: Plants (Basel). 2024 Apr 3;13(7):1023. doi: 10.3390/plants13071023 (PMC11013679; doi:10.3390/plants13071023)
Supplement: Supplementary file 1 [file plants-13-01023-s001.zip › plants-2801212-supplementary.pdf]

Table S1. Mean performance of *E. procerus* derived hybrids and standard varieties during the year 2018-19

Clones	Tiller 60 DAP (000-ha)	Tiller 90D AP (000-ha)	Tiller 120 DAP (000-ha)	PH (cm)	CD (cm)	JW (kg)	JE (%)	Brix (%)	Pol % (%)	Pur (%)	SCW (kg)	Fibre (%)	NMC (000-ha)	CY (t/ha)	NBI	Chl (µg/cm²)	Flav	Anth	LA (cm²)
**Co 0238**	146.67	150.26	165.19	233.33	2.47	2.58	47.34	20.51	18.21	88.84	1.08	14.19	87.41	92.69	23.83	28.41	1.20	0.18	203.12
**Co 06027**	88.89	84.44	83.70	186.67	2.97	3.16	53.70	20.56	17.95	87.28	0.92	14.70	75.37	69.55	33.03	38.70	1.18	0.13	277.25
**CoJ 64**	112.59	126.67	129.63	140.00	1.87	1.06	40.16	20.32	17.98	88.49	0.47	14.82	97.04	44.06	21.27	29.11	1.38	0.12	273.02
**CoS 767**	108.89	125.93	132.59	136.67	1.83	0.97	39.58	19.71	16.57	84.01	0.58	14.57	81.85	47.09	30.01	37.02	1.24	0.15	256.21
**CoS 8436**	110.37	104.44	140.00	190.00	2.23	1.50	37.27	19.36	16.81	86.62	0.65	13.54	96.30	63.61	28.69	34.61	1.22	0.15	237.64
**GU 04(28)EO-2**	127.37	103.70	171.85	243.33	1.50	0.27	12.29	13.68	9.14	66.65	0.35	21.34	102.22	35.15	38.04	46.84	1.16	0.12	191.72
**GU 12-16**	137.04	129.63	148.89	220.00	1.80	0.98	28.87	17.16	14.75	85.67	0.65	20.59	102.96	65.23	33.37	38.63	1.15	0.15	269.72
**GU 12-19**	191.11	219.26	261.48	226.67	1.87	1.18	31.13	17.27	14.74	85.19	0.58	22.46	130.37	72.90	41.23	38.90	0.95	0.13	213.01
**GU 12-21**	93.33	99.56	120.00	193.33	2.00	0.84	30.89	15.20	12.23	80.20	0.58	21.13	134.81	77.40	35.54	37.73	1.05	0.14	223.67
**GU 12-22**	67.04	81.00	97.78	146.67	2.43	0.94	36.59	16.08	13.26	82.42	0.68	19.84	92.59	62.70	26.63	29.43	1.11	0.18	254.41
**GU 12-23**	102.78	100.37	114.44	213.33	2.00	1.27	36.33	16.61	14.34	85.34	0.87	19.45	83.70	72.38	31.89	38.78	1.26	0.15	236.33
**GU 12-26**	131.85	157.78	197.04	253.33	1.97	1.50	37.08	18.66	16.28	87.24	0.75	17.96	92.22	68.37	33.64	39.53	1.17	0.16	185.56
**GU 12-27**	169.63	174.81	155.56	263.33	2.07	1.59	35.43	16.36	13.97	85.33	0.62	17.93	120.74	74.92	34.99	34.50	1.01	0.15	220.28
**GU 12-28**	169.63	167.41	198.00	243.33	1.83	0.99	30.71	16.64	12.51	75.49	0.81	24.20	101.48	81.40	37.51	39.89	1.07	0.14	244.35
**GU 12-29**	102.96	132.59	220.00	156.67	1.67	0.77	31.26	16.04	11.78	72.93	0.46	22.34	130.44	57.46	30.26	35.88	1.19	0.16	254.40
**GU 12-30**	210.00	220.59	182.96	243.33	1.73	1.03	31.68	16.51	13.90	84.27	0.89	24.92	110.67	91.88	20.72	25.73	1.27	0.16	233.55
**GU 12-31**	158.52	146.67	147.33	233.33	2.07	1.24	34.71	15.85	13.34	84.10	0.66	22.47	119.85	78.58	36.48	41.84	1.16	0.14	303.59
**GU 12-33**	131.11	149.63	115.56	193.33	1.70	0.73	29.30	15.96	13.15	81.98	0.54	22.94	113.33	61.76	41.93	36.70	0.89	0.13	247.83
**GU 12-34**	105.93	115.56	159.78	173.33	2.13	1.16	36.77	18.42	15.69	85.45	0.62	19.92	111.11	68.17	35.12	36.26	1.04	0.14	271.68
**GU 12-38**	173.33	169.63	160.74	223.33	2.00	1.63	40.66	18.31	16.18	88.48	1.03	23.94	95.19	99.16	27.42	31.47	1.15	0.15	263.98
**GM**	131.95	138.00	155.13	205.67	2.01	1.27	35.09	17.46	14.64	83.30	0.69	19.66	103.98	69.22	32.08	36.00	1.14	0.15	243.07
**Minimum**	67.04	81.00	83.70	136.67	1.50	0.27	12.29	13.68	9.14	66.65	0.35	13.54	75.37	35.15	20.72	25.73	0.89	0.12	185.56
**Maximum**	210.00	220.59	261.48	263.33	2.97	3.16	53.70	20.56	18.21	88.84	1.08	24.92	134.81	99.16	41.93	46.84	1.38	0.18	303.59
**std**	36.34	38.46	41.63	38.28	0.32	0.63	7.94	1.88	2.29	5.52	0.19	3.56	16.61	15.64	5.85	4.96	0.11	0.01	29.71

PH- Plant height, CD- cane diameter, JE-Juice extraction, , Pol%- Sucrose % in juice, SCW- Single cane weight, CY- Cane yield, NBI-Nitrogen balance index, Chl-Chlorophyll concentration, Falv- Falvonoids index, Anth- Anthocaynin index and LA- leaf area, GM-Grand Mean, std- standard deviation.

Table S2. Mean performance of *E. procerus* derived hybrids and standard varieties during the year 2019-20

Clones	Tiller 60 DAP (000-ha)	Tiller 90DAP (000-ha)	Tiller 120 DAP (000-ha)	PH (cm)	CD (cm)	JW (kg)	JE (%)	Brix (%)	Pol % (%)	Pur (%)	SCW (kg)	Fibre (%)	NMC (000-ha)	CY (t/ha)	NBI	Chl (μg/cm²)	Flav	Anth	LA (cm²)
**Co 0238**	140.00	70.00	58.15	238.33	2.35	2.41	45.22	18.84	16.17	85.88	1.08	22.84	87.31	90.50	29.34	38.41	1.33	0.13	274.55
**Co 06027**	88.89	82.96	70.00	182.67	2.84	3.06	52.21	19.46	16.83	86.59	0.49	16.24	117.04	47.59	29.49	33.20	1.14	0.14	223.00
**CoJ 64**	112.59	69.26	63.33	142.00	1.47	1.26	43.21	18.41	15.70	85.22	0.47	11.53	97.04	44.06	19.38	28.80	1.52	0.15	217.64
**CoS 767**	108.89	80.37	64.44	137.67	1.89	0.99	34.21	18.70	16.00	85.54	0.59	14.42	78.52	46.47	31.38	37.20	1.19	0.14	251.54
**CoS 8436**	110.37	69.63	57.78	184.74	2.03	1.47	37.21	17.86	14.88	83.24	0.72	13.83	96.30	72.89	23.71	35.40	1.49	0.15	213.30
**GU 04(28)EO-2**	79.26	67.78	61.11	240.30	1.54	0.29	15.34	14.85	11.36	76.34	0.38	21.81	102.22	38.12	40.60	44.30	1.10	0.14	247.42
**GU 12-16**	137.04	66.67	64.07	120.12	1.78	0.94	29.15	16.21	12.83	79.16	0.66	19.23	102.96	66.03	32.99	35.11	1.07	0.13	259.87
**GU 12-19**	191.11	85.19	85.56	230.47	1.97	1.08	33.15	18.23	15.51	85.05	0.58	17.89	130.37	72.90	38.83	36.19	0.93	0.12	205.53
**GU 12-21**	80.00	92.59	77.78	173.13	2.01	0.88	28.66	15.52	12.71	81.77	0.58	20.36	134.81	77.40	33.63	37.17	2.16	0.16	218.62
**GU 12-22**	50.37	75.56	67.41	136.87	2.33	0.92	34.84	16.01	12.69	79.04	0.49	16.92	99.26	48.79	21.46	28.84	1.36	0.15	237.07
**GU 12-23**	77.78	54.81	52.59	253.43	1.88	1.21	34.15	16.34	13.10	79.69	0.85	19.20	83.70	71.23	41.73	40.08	0.97	0.13	213.20
**GU 12-26**	131.85	65.93	61.85	248.37	1.87	1.59	35.15	18.00	15.27	84.77	0.74	18.92	77.22	56.04	33.92	40.98	1.20	0.16	234.89
**GU 12-27**	169.63	90.15	97.41	263.33	2.17	1.46	31.44	15.64	12.58	80.21	0.72	18.86	102.22	73.87	30.27	33.56	1.11	0.14	218.03
**GU 12-28**	169.63	75.56	60.74	242.33	1.73	1.09	30.21	15.37	11.71	76.29	0.77	18.74	82.74	63.39	37.33	40.49	1.11	0.13	253.28
**GU 12-29**	102.96	63.70	62.96	159.47	1.47	0.79	33.48	17.13	13.54	78.28	0.43	20.42	120.44	43.97	24.31	30.44	1.25	0.15	226.00
**GU 12-30**	200.00	93.70	83.96	247.35	1.83	1.01	30.22	16.77	13.54	80.92	1.15	21.60	81.03	92.85	20.14	25.58	1.27	0.17	208.62
**GU 12-31**	158.52	104.07	99.63	231.32	2.14	1.20	34.21	15.40	12.23	79.30	0.64	18.85	109.52	68.03	27.14	30.29	1.12	0.16	284.53
**GU 12-33**	97.78	61.11	62.85	190.23	1.08	0.63	30.55	16.65	14.00	83.62	0.65	22.75	101.00	62.14	32.10	32.76	1.04	0.14	216.37
**GU 12-34**	72.59	76.30	66.67	172.33	2.24	1.16	35.14	16.74	13.40	79.79	0.51	17.05	111.11	60.09	38.38	39.46	1.03	0.11	269.70
**GU 12-38**	173.33	100.56	87.78	220.33	1.94	1.63	39.55	17.61	15.17	85.90	1.04	17.11	87.19	90.03	24.71	27.93	1.14	0.16	270.72
**GM**	122.63	77.29	70.30	200.74	1.93	1.25	34.37	16.99	13.96	81.83	0.68	18.43	100.11	64.32	30.54	34.81	1.23	0.14	237.19
**Minimum**	50.37	54.81	52.59	120.12	1.08	0.29	15.34	14.85	11.36	76.29	0.38	11.53	77.22	38.12	19.38	25.58	0.93	0.11	205.53
**Maximum**	200.00	104.07	99.63	263.33	2.84	3.06	52.21	19.46	16.83	86.59	1.15	22.84	134.81	92.85	41.73	44.30	2.16	0.17	284.53
**Std**	42.17	13.23	13.22	10.26	0.09	0.14	1.65	1.31	1.57	3.26	0.21	2.87	16.32	16.12	6.64	4.94	0.26	0.02	24.21

PH- Plant height, CD- cane diameter, JE-Juice extraction, , Pol%- Sucrose % in juice, SCW- Single cane weight, CY- Cane yield, NBI-Nitrogen balance index, Chl-Chlorophyll concentration , Falv- Falvonoids index, Anth- Anthocaynin index and LA- leaf area, GM-Grand Mean, std- standard deviation.
